# Word Category Conversion Revisited: The Case of Adjectives and Participles in L1 and L2 German

**DOI:** 10.3389/fpsyg.2020.01045

**Published:** 2020-05-28

**Authors:** Andreas Opitz, Denisa Bordag

**Affiliations:** ^1^Herder Institute, Leipzig University, Leipzig, Germany; ^2^University of Haifa, Haifa, Israel

**Keywords:** conversion, mental lexicon, priming, frequency, participle

## Abstract

One of the hypotheses about mental representation of conversion (i.e., zero-derivation) claims that converted forms are a product of a costly mental process that converts a word’s category into another one when needed, i.e., depending on the syntactic context in which the word appears. The empirical evidence for the claim is based primarily on self-paced reading experiments by Stolterfoht et al. (2010) in which they explored the assumed conversion of German verbs into adjectives in two syntactic contexts with past participles. In our priming study, we show that the effects that had been attributed to the conversion process are in fact frequency effects. In addition, based on our data we argue that past participles do not undergo any change in word class in either of the two syntactic contexts, which is consistent with, e.g., traditional German grammars. The same pattern of frequency effects was observed for German native speakers and advanced L2 German learners.

## Introduction

Word class category information is crucial for constructing syntactic representations and language comprehension in general. According to lexicalist approaches, which we address in this paper, this information is stored for each word in the mental lexicon (Chomsky, 1970; Levelt, 1999). While in some languages (like German or Czech), word class specific inflectional marking typically enables word class assignment even to isolated word forms, sometimes word forms can be ambiguous as it is often the case in English, where it is the syntactic context that determines the word class, e.g., *Mary is surprising_VERB_ us*, vs. *Mary told us a surprising_ADJ_story.*

Interestingly, there is only sparse psycholinguistic research concerning the processing of category ambiguous forms (e.g., Pliatsikas et al., 2014; Lukic et al., 2019) and to our knowledge no study that would compare their processing in L1 and L2. One of the few studies is by Stolterfoht et al. (2010) who argue in favor of a lexicalist account that involves a productive category changing procedure converting past participle verb forms into adjectives when needed. The study basically delivers the only psycholinguistic evidence for the representation and processing of conversion forms (also called zero-derivation) by means of a productive process (cf. Bauer and Valera, 2005, for other empirically based proposals of conversion representation). It is also the only psycholinguistic study that addresses the putative word-class change of past participle forms in German. Since both the claim that past participles are processed as adjectives in certain passive contexts and that conversion forms are a result of a productive process were based on just one experiment, we considered it desirable to address the same research questions with a different paradigm (grammaticality judgment task with a priming component) and to test whether the same processing mechanisms are employed by native German speakers and advanced L2 German learners with L1 Czech. As we show, our results favor an explanation based on frequency effects and shed doubt on the assumption that depending solely on the syntactic position, German past participles are processed either as verbs or as adjectives.

## Study of Stolterfoht et al. (2010)

Stolterfoht et al.’s study is grounded in the assumption that there is a “verbal” and an “adjectival” passive in German, which both contain a morphologically ambiguous form of a past participle (example sentences based on Stolterfoht et al., 2010):

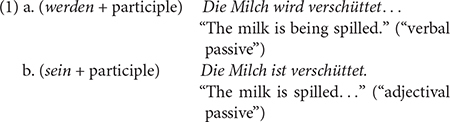

This assumption is, however, a controversial topic that has been extensively discussed in the linguistic literature (cf. Roy et al., 2013, for an overview). Some accounts share Stolterfoht et al.’s view (e.g., Rapp, 1997; Von Stechow, 1998; Kratzer, 2000; Maienborn, 2007), usually based on semantic considerations. In contrast, the majority of traditional accounts (Dudenredaktion, 2006; Helbig and Buscha, 2017) assume that participles are non-finite verb forms in both passive constructions and that the two examples differ only in that (1a) expresses a process (“Vorgangspassiv,” i.e., “procedural passive”) or (1b) its result (“Zustandspassiv”, i.e., “stative passive”). Adjectival status is assigned to participles only if they fulfill certain morphosyntactic criteria, i.e., conform to adjectival declension (i.e., inflectional marking of case, number, and gender, e.g., *ein geschlossen-es Fenster* “a closed(NOM/ACC.SINGULAR.NEUTER) window”).

Adopting the view that the past participle is a verb in (1a) and an (converted) adjective in (1b), Stolterfoht et al. argue within a lexicalist approach that the lexically specified category information of *verschüttet* (verb) must be converted into another category (adjective), and that this additional process of conversion leads to additional processing costs measurable in longer reading times. While this is one possible implementation of lexicalist accounts, it is by far not exhaustive. Lexical entries, for instance, may have an internal structure with word class specific sub-entries. According to such approaches, derived categories (e.g., adjective in the present case) are nested as sub-nodes under the main node from which they are derived (e.g., verb node). No supplementary process of category conversion is thus needed, only different (sub)parts of one lexical entry need to be accessed (cf. Bauer and Valera, 2005). On the other hand, in syntactic approaches (e.g., Borer, 1994; Marantz, 1997) that Stolterfoht et al. do not address experimentally, the syntactic context determines the word category and verbal and adjectival forms are derived from a category-neutral root by adjoining a category head. In such a scenario, processing efforts should be equally costly.

In their self-paced reading experiment, Stolterfoht et al. compared reading times of ambiguous (de)verbal forms (e.g., *verschüttet*) with those of genuine adjectives presented in the same syntactic contexts. They hypothesized that while processing should be the same for genuine adjectives that have the same word class category in both contexts (2a vs. 2b), the processing of participles involves a category change in adjectival contexts (1b), but not in verbal contexts (1a). The higher processing costs of the category-changing procedure should be manifested in slower reading times in (1b).

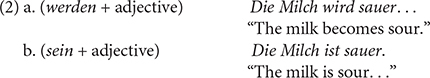

These were indeed the results that Stolterfoht et al. obtained for participles: significantly faster reading times in verbal than in adjectival contexts. No such difference was observed for adjectives. The authors interpret their findings as evidence for a lexicalist interpretation including a productive conversion process.

However, there is a caveat in this explanation, namely the frequency of co-occurrences. Participle forms are more frequent after *werden* (1a) than after *sein* (1b). Adjectives, on the other hand, occur more frequently after *sein* than after *werden* (cf. the corpus analysis reported in Stolterfoht et al., 2010: Table 1). Due to probabilistic expectancies of the parser, a verbal form is less surprising after *werden* and an adjective form after *sein*. A frequency-based account would thus predict slower reading times on participles after *sein* without the necessity of postulating any conversion process. Crucially, it would also predict slower reading times on adjectives after *werden*. Stolterfoht et al. argue that since they do not find such effects for adjectives, it is thus the costly conversion process that is responsible for the difference in the reading times in the participle condition.

It should be however, noted that (a) there was a numerical tendency of 15 ms in the expected direction based on frequency in the genuine adjective condition (compared to 33 ms for participles); (b) for the items used in the experiment (see Stolterfoht et al., 2010, Table 2), the relative difference in terms of co-occurrence between *werden* vs. *sein* was more than twice as large for participles (1:5.4) than for adjectives (2.2:1) running against the overall pattern that for adjectives the differences between the two contexts is generally more pronounced; the skewed item selection might have contributed to the observed null-effect for adjectives; and (c) the crucial null-effect for adjectives coincided with large *SD*s and a rather small sample size of items (*N* = 12) indicating low statistical power.

Obviously, more robust data are necessary to support the lexicalist conversion process hypothesis, or to deliver stronger evidence for or against alternative explanations (e.g., frequency-based).

## The Present Study

We tested the same lexicalist hypothesis as formulated by Stolterfoht et al., namely that there is a morphosyntactic process that converts verbal participle forms into adjectives when they appear in particular syntactic contexts. We designed the experiment such that it also assesses an alternative lexicalist account assuming that conversion forms are represented as subnodes of a basic entry (cf. Bauer and Valera, 2005). Therefore, we used a grammatical decision task combined with priming. In order to avoid the caveats of Stolterfoht et al.’s study, we used more and better controlled items that followed the general trends for co-occurrences of adjectives/participles with *sein/werden*.

We also compared native and non-native processing. There are two main views regarding the differences in processing of morphologically complex words in L2 (Kırkıcı and Clahsen, 2013, p. 778). According to the first view (e.g., McDonald, 2006), processing mechanisms are fundamentally the same as in L1 and the differences arise only due to the fact that L2 processing is slower, cognitively more demanding and affected by L1. The second view states that there are differences in the processing mechanisms themselves, in that, for example, the L2 mechanism works in a “shallower” manner (e.g., Clahsen and Felser, 2006 see also Ullman, 2005). Accordingly, L2 learners should be less likely to engage an additional morphosyntactic operations (conversion) compared to native speakers. In contrast to such types of processing differences, frequency-based processing differences are typically observed in both L1 and L2^1^. Thus, comparing native and non-native morphosyntactic processing can potentially help to differentiate between the two views and to advance our understanding of the nature of L2 processing.

While in Stolterfoht et al. (2010) the critical forms were embedded in sentences, the critical items in our study (genuine adjectives and participles) were presented as continuations of minimal syntactic contexts that involved the disambiguating verbs *werden* and *sein*.

The syntactic context was kept minimal in order to reduce lexically based expectations. Participants made grammaticality decisions over the phrases at the presentation of the critical word. We hypothesized (Hypothesis A1) that if a conversion process is involved for participles in the *sein*-context, processing should be more demanding in these cases and evidenced in longer response latencies (cf. Stolterfoht at al.). On the other hand (Hypothesis A2), if the results of Stolterfoht et al. were artifacts of frequency effects, we expected that reaction times would ally with the frequency of co-occurrences of the minimal contexts with adjectives and participles.

**FIGURE 1:**
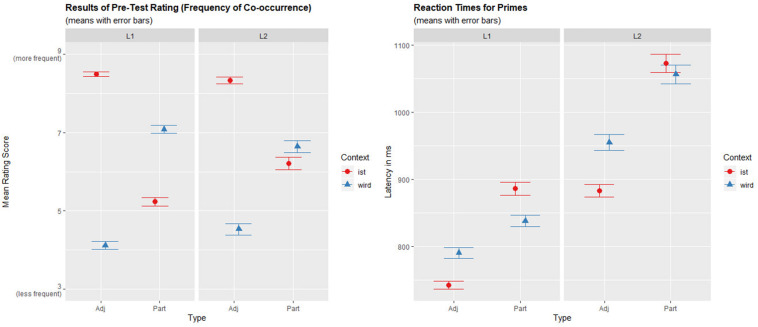
Comparison of the results of the rating (on the left) with the reaction times to prime phrases (on the right) showing a correspondence between the rating scores and the RTs: Contexts that were rated more frequent (*ist* + adjective and *wird* + participle) were responded to faster. The differences between *ist/wird*-contexts were significant for adjectives both in L1 and L2, while for participles they were only significant in L1 (*p* = 0.003 both for ratings and RTs), but not for L2 (*p* = 0.353 for ratings and *p* = 0.301 for RTs) (Mixed effects models: Score/RT ∼ Language × Type × Context + (1 + Context × Type | Participant.ID) + (1 + Language × Context | Item.ID)).

In order to obtain valid and comparable frequency measures for both L1 and L2 (which have different input frequency), we conducted a rating in which samples from both populations judged on a 10-point-scale how frequent was the appearance of a given item within either a *sein*- or a *werden*-context. The L1-results (*n* = 42) corresponded to the overall frequencies reported by Stolterfoht et al. (2010) in Table 1: Co-occurrences of *werden* + participles and *sein* + adjectives were judged more frequent than the alternate combination, and the difference was larger for adjectives than for participles. The L2-data (*n* = 17) differed in that there was no difference for the participles between the two contexts (see Figure 1).

For the priming component, the critical form was repeated either in the same or in a different (*werden/sein*) syntactic context. Adjectives and participles were thus both presented in four different priming conditions, see Table 1.

**TABLE 1:** Examples of items in each of the experimental conditions.

Word Category	Prime		Target		Context relation of prime-target
		
	Context	Item	Context	Item	
Adjectives	“he is/becomes jealous”				
	*er ist*	*neidisch*	*er ist*	*neidisch*	Same
	*er wird*	*neidisch*	*er ist*	*neidisch*	Different
	*er wird*	*neidisch*	*er wird*	*neidisch*	Same
	*er ist*	*neidisch*	*er wird*	*neidisch*	Different
Participles	“he is/is being destroyed”				
	*er ist*	*zerstört*	*er ist*	*zerstört*	Same
	*er wird*	*zerstört*	*er ist*	*zerstört*	Different
	*er wird*	*zerstört*	*er wird*	*zerstört*	Same
	*er ist*	*zerstört*	*er wird*	*zerstört*	Different

*There were also control conditions with unrelated adjectives/participles in prime phrases that are left out here for clarity reasons.*

While there is an extensive priming research involving stems and derived forms (mostly indicating bi-directional priming between the two, e.g., *govern – government*, but no priming between derived forms like *govern-ment* vs. *govern-or*) (see Marslen-Wilson, 2007 for an overview), little is known about the processing of zero-derived words with a different word-class status. Previous research on noun-verb conversion in German using analogical design (Bordag and Opitz, under revision) revealed reduced priming for form-identical prime-target pairs that belong to different word classes (verbs vs. nouns as conversion products) compared to form-identical pairs within the same category (verbs with different morphosyntactic features). For instance, the target phrase *wir MIETEN* (“we rent”) showed full priming after the prime phrase *sie MIETEN* (“they rent”), but only partial priming after the conversion form phrase *das MIETEN* (“the renting”), despite the formal identity and the close semantic relation between the two words. Such findings indicate that processing of conversion products involves different lexical entries (possibly subentries within a shared base-entry), specified for word category. We thus hypothesized (Hypothesis B) that if adjectival and verbal participles were represented according to this version of a lexicalist approach, we would observe reduced priming effects in the changed context condition (*wird* > *ist; ist* > *wird*, i.e., accessing different (sub)nodes) compared to the same context condition (*wird* > *wird, ist* > *ist*) for participles, but not for genuine adjectives. Including a priming component thus extends the potential to tease apart frequency-based effects from effects originating in different lexical representations.

### Methods

#### Participants

All participants gave written informed consent in accordance with the Declaration of Helsinki and were paid for their participation. None of them participated in the rating study.

***L1.*** Seventy-two (18M, 54F) German native speakers, aged 18 to 38 years (24.4 on average, *SD* = 8.2) were tested.

***L2.*** The ages of the 60 (9M, 51F) Czech natives ranged between 18 and 35 years (22.7 on average, *SD* = 3.18). Their German proficiency was assessed using three different measures: a version of the Goethe Test, an online version of DiaLang^2^, and a self-evaluation questionnaire. Only participants that reached C1 level in at least one test and not less than B2 level in the other tests qualified for the experiment. The advanced level of proficiency guaranteed that they were familiar with all tested structures.

#### Materials

Thirty German adjectives and 30 participles were selected as items. Morphological formation of the participle was balanced (half of them with prefixation of *ge*-, the other half without that prefix). A corresponding group of 30 adjectives were chosen such that they were pairwise matched with the participles with respect to word length [mean number of letters: participles = 8.6 (sd = 1.3), adjectives = 8.6 (sd = 1.5)] and frequency class [assessed via Wortschatz-Project of Leipzig University, means: participles = 10.8 (sd = 2.12), adjectives = 10.9 (sd = 2.08)]. A list of all experimental items can be found in the Supplementary Material.

Items were distributed over five different lists such that no item was repeated. Each list contained all 30 adjectives and 30 participles once in one of the four conditions (see Table 1). All conditions were counter-balanced across lists (Latin Square design). The order of items was pseudo-randomized for each participant.

All experimental trials were pairs of a prime phrase immediately followed by a target phrase sharing an identical word form (adjective/participle). All of them were grammatical. Additionally, a large number of filler phrases was created, also including primed (i.e., repeated) filler items (paralleling the presentation of critical items), but some of them were ungrammatical. There were always at least three filler trials between experimental trials. The whole design was completely cross balanced for correct/incorrect forms, item repetition, and type of syntactic structure in order to avoid strategic effects or probability-based confounds. Ungrammatical fillers included incorrect agreement marking with respect to number, person, or gender (e.g., *mit vielen Wolke* “with many cloud(SINGULAR)”). In total, each experimental list consisted of 672 single judgment tasks (60 × 2 experimental trials + 552 fillers).

#### Procedure

In written instructions, participants were familiarized with the task and instructed that they should respond as fast and accurate as possible. L2 participants also took language tests. The experimental stimuli were presented using the E-Prime 2.0 software (Psychology Software Tools, Pittsburgh, PA, United States).

All trials, including primes, targets, and fillers were presented following an identical procedure: After a fixation sign (“×”) was presented at the center of the screen for 500 ms, a phrase was displayed in two stages. First, all material preceding the participle or adjective, i.e., the context, was presented centered on the screen (e.g., *er ist* “he is”). After 750 ms these words disappeared and the second part of the phrase (participle or adjective) was presented in capital letters at the same position. Participants were instructed to only respond to the part in capital letters and to judge whether it is a grammatical or ungrammatical completion of the phrase by pressing one of two corresponding buttons. Participants responded to both primes and targets (and filler trials). After the participant’s response or after a maximum duration of 2000 ms, the word disappeared from the screen. At the beginning of the experiment, there was a training block to familiarize participants with the task. An average experimental session took about 40 (L1) and 45 (L2) minutes.

### Results and Discussion

#### Data Preparation and Analyses

Response latencies that deviated by more than three standard deviations from a participant’s mean were considered outliers and excluded from further analyses (1.29% of prime responses, 0.91% of target responses).

**FIGURE 2:**
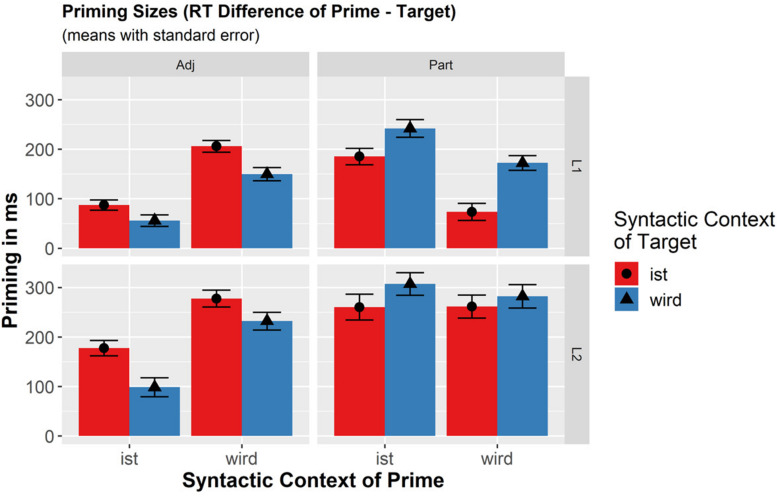
Effects for priming sizes were computed by the difference of reaction times to primes and corresponding target phrases (RT.Prime−RT.Target = RT.Diff). The formula of the final mixed effects model for Analysis B (priming sizes) was: RT.Diff ∼ Language × Context.Prime × Context.Target + (1 + Context.Prime × Context.Target| Participant.ID) + (1 + Language × Context.Prime × Context.Target | Item.ID).

All analyses were performed using linear mixed-effect models employing the software *R* (R Core Team, 2018) with package *afex* (Singmann et al., 2020) (with Satterthwaite and Kenward-Roger methods for denominator degrees of freedom for *t* and *F* tests). All models included random intercepts for participants and items and random slopes for all independent variables and their interactions (cf. formulas in captions to Table 2 and Figure 2).

**TABLE 2:** Mean reaction times to prime phrases in ms (accuracy in %).

	Participles		Adjectives		
		
	ist	wird	ist	wird	Mean
L1 German	886.0 (82.7%)	839.8 (90.0%)	737.5 (98.3%)	788.4 (91.7%)	*812.9 (90.7%)*
L2 German	1067.4 (81.6%)	1061.3 (87.6%)	876.8 (97.9%)	944.6 (91.6%)	*987.5 (89.7%)*
*Mean*	*976.7 (82.2%)*	*950.6 (88.9%)*	*807.2 (98.2%)*	*866.5 (91.6%)*	*900.2 (90.2%)*

*The formula of the final mixed model for Analysis A (reaction times to prime phrases) was: Prime.RT ∼ Language × Type × Context + (1 + Type × Context | Participant.ID) + (1 + Language × Context | Item.ID).*

#### Analysis A

Response latencies to the first occurrences of the critical items were analyzed (i.e., those that were primes in the priming component of the experiment) to assess Hypothesis A. Only correct responses entered the analysis (data loss = 9.72%). We also analyzed accuracy rates, but do not report them here for reasons of clarity and space. However, results fundamentally mirrored those of reaction times: shorter latencies were accompanied systematically by higher accuracy rates (see Table 2).

In general, L1 participants were faster than L2 participants [β = −93.55, *SE* = 12.46, *t*(142.28) = −7.51, *p* < 0.0001]. The main effect for Type [β = −64.50, *SE* = 9.30, *t*(72.30) = 6.933, *p* < 0.0001] revealed that participants were significantly slower when responding to participles than to adjectives. The significant interaction Type:Language [β = 15.41, *SE* = 5.46, *t*(93.40) = 2.82, *p* = 0.006] and its subsequent resolution showed that the effect was larger in L2 [β = −167.45, *SE* = 24.11, *t*(80.34) = −6.95, *p* < 0.0001] than in L1 [β = 107.9, *SE* = 13.33, *t*(64.56) = −7.56, *p* < 0.0001].

The influence of Context (*ist/wird*) was not significant [β = −7.16, *SE* = 4.36, *t*(67.45) = −1.64, *p* = 0.105]. A significant interaction of Context:Type [β = −25.19, *SE* = 4.12, *t*(63.67) = −6.11, *p* < 0.0001] was resolved revealing that while *wird*-contexts led to slower reaction times than *ist*-contexts for adjectives [β = 60.95, *SE* = 10.26, *t*(33.09) = 5.94, *p* < 0.0001], for participles, this pattern was reversed: reaction times were shorter for *wird*-phrases than for *ist*-phrases [β = −38.54, *SE* = 12.97, *t*(28.27) = −2.97, *p* = 0.006]. Figure 1 visualizes the interaction of Type:Context together with the results of the rating.

The results do not support Hypothesis A1 claiming that a costly conversion process is responsible for the slower reaction times to participles in the *ist*-context. Crucially—and in contrast to Stolterfoht et al.—we do find analogous differences also for genuine adjectives. The results exactly correspond to a pattern expected by frequency-based accounts (Hypothesis A2): slower reaction times for less frequent contexts (*sein* for participles and *werden* for adjectives). This interpretation is further substantiated when frequency (i.e., means from the rating for each item in the two contexts) is included in the analysis as a covariate: The resulting model is a better fit than the original model [AIC = 77508 vs. 77488, Chi2(1) = 21.6, *p* < 0.001]. Moreover, while the effects of Type (*p* < 0.001) and Language (*p* < 0.0001) remain significant, the interaction Type:Context does not survive (*p* = 0.446), indicating that the formerly observed influence of Context is better explained by Frequency (*p* < 0.001).

#### Analysis B

Priming effects were analyzed in order to assess whether different representations are accessed in so-called verbal versus adjectival contexts for participles (Hypothesis B). Only correct responses to participles were analyzed (data loss = 4.51%). According to Hypothesis B, priming size should be reduced in the “changed” context condition compared to the “identical” context condition.

In general, priming was larger in L2 than in L1 [278 ms vs. 168 ms; β = −59.25, *SE* = 11.61, *t*(68.37) = −5.10, *p* < 0.0001] and for the target *wird*-contexts than for *ist*-contexts [251 ms vs. 195 ms; β = −28.66, *SE* = 8.72, *t*(40.48) = −3.29, *p* = 0.002]. There was also a main effect of Priming Context revealing that priming size was larger for the prime *ist*-contexts than *wird*-contexts [249 ms vs. 197 ms β = 27.26, *SE* = 10.68, *t*(27.14) = 2.55, *p* = 0.017]. A significant interaction of Prime Context and Language [β = 17.67, *SE* = 8.09, *t*(39.65) = 2.19, *p* = 0.035] indicated that the difference between *ist*- vs. *wird*-prime contexts was significant only in L1 [β = 45.69, *SE* = 13.88, *t*(29.86) = 3.29, *p* = 0.003], but not in L2 [β = 9.74, *SE* = 12.19, *t*(59.84) = 0.799, *p* = 0.427].

Crucially, neither the interaction of Prime Context with Target Context [β = 3.89, *SE* = 8.55, *t*(34.51) = 0.46, *p* = 0.651], nor the triple interaction with Language [β = 7.81, *SE* = 7.49, *t*(40.27) = 1.04, *p* = 0.303] were significant. Thus, there were no indications that certain combinations of prime and target contexts lead to differences in priming sizes in any of the populations. In particular, with respect to Hypothesis B, it was not the case that the same contexts in primes and targets (*wird-wird* and *ist-ist*: mean 225.1 ms) would lead to larger priming sizes than changed contexts (*wird-ist* and *ist-wird*: mean 221.2 ms).

Figure 2 illustrates the findings, additionally showing the results for the control group of adjectives. Its statistical analyses are left out here due to space limitations and irrelevance to the research question. However, in parallel to participles, also for adjectives there are main effects of Language, Target Context, and Prime Context, but none of the interactions turned out to be significant (all *p*-values > 0.25). Most importantly, the influence of Prime and Target Contexts for the adjectives was completely reversed compared to the effects for participles in both L1 and L2. For adjectives, larger priming was observed for *ist*-contexts in target phrases and *wird*-contexts in primes.

## General Discussion

In the present study, we explored the representation and processing of German participles in predicate position, following either the verb *sein* or *werden* in L1 and L2 German. This research question was previously addressed by Stolterfoht et al. (2010) who claimed that German participles were turned into adjectives by a conversion process in the *sein*-contexts. We tested this claim (Hypothesis A1) employing a method more sensitive to grammatical processing and using more and better controlled items and observed significant processing differences between the contexts for both participles and adjectives. Both classes are processed faster in their more frequent context (in prime and in target phrases), and they are primed better when the preceding phrase contains their less frequent syntactic context (*werden* for adjectives, *sein* for verbs). The pattern of results reveals no indications of any conversion process and is best compatible with frequency-based accounts that predict slower processing in less frequent/preferred contexts (Hypothesis A2).

However, the frequency of contexts also corresponds to prototypical meanings of adjectives and participles: While adjectives typically denote stative properties (compatible with the semantics of the verb *sein* “to be”), participles (as verb forms) denote actions or processes (compatible with semantics of the verb *werden*). The factors frequency and prototypicality of meaning are thus confounded and probably both contribute to the observed processing differences.

We also tested an alternative lexicalist hypothesis (B) that converted forms are stored as subnodes within the main entry of a word using a priming paradigm (Bauer and Valera, 2005; Author, submitted). This hypothesis was not supported by our data, either. We found no evidence that participles would have a different word class status in *werden*- vs. *sein*-contexts: In all analyses, participles behaved exactly like adjectives for which no word class change in the two contexts is expected—but in a reversed pattern that corresponds to their frequency of cooccurrences with the *sein/werden* verbs. Our results thus do not support any of the two lexicalist accounts tested in the present study, but they are compatible with traditional German grammars that view past participles in both contexts as verbal forms. More research is clearly necessary to test also the alternative hypotheses, e.g., those based on the syntactic accounts. The results of our priming study do not indicate that participles would surface once as adjectives and once as verbs in the two contexts (while having the same category-neutral root representation), but the employed method may not be suitable to test such hypothesis.

Moreover, also the interpretation of the exact pattern of the priming results would require more speculation and research, e.g., exploring access to and processing of central/frequent portions of semantic space of a specific word class vs. its more peripheral/less frequent portions (cf. research on hypernym/hyponym relations, e.g., Sharifian, 2002; Crossley, 2013).

The comparison of L1 speakers and advanced L2 learners revealed that despite numerical differences indicating that the L2 processing is not fully native-like, statistically, both populations were similarly sensitive to context manipulations of primes and targets. Since the original morphosyntactic hypothesis about conversion was not validated, the current results cannot be used in support of any hypothesis about L2 morphosyntactic processing. However, they reveal comparable sensitivity to frequency effects and comparable representation of adjectives and participle verb forms in L1 and L2 German, highlighting the role of this factor for psycholinguistic research. To conclude, the results of the present study highlight the need of more L1 and L2 psycholinguistic research investigating the mental status of participles and zero-derived forms in general.

## Data Availability Statement

The datasets generated for this study are available on request to the corresponding author.

## Ethics Statement

This study was carried out in accordance with the recommendations of DFG (German Research Council). The protocol was approved by the DFG. Further ethical review and approval was not required for the study on human participants in accordance with the local legislation and institutional requirements. The participants provided their written informed consent to participate in this study.

## Author Contributions

Both authors contributed to the preparing, programming, running of the experiments as well as to data analyzing and reporting the results of the experiments to equal parts.

## Conflict of Interest

The authors declare that the research was conducted in the absence of any commercial or financial relationships that could be construed as a potential conflict of interest.

## Footnotes

1 In Czech, the corresponding difference is not expressed through different auxiliaries, but through different aspect (*pokus je provádìn* (imp.)– “experiment is being performed” vs. *pokus je proveden* (perf.) “experiment is performed,” or alternatively, with a reflexive form *pokus se provádí* for the process meaning).

2 Public access via: https://dialangweb.lancaster.ac.uk/
