# Supplementary material for: Word Category Conversion Revisited: The Case of Adjectives and Participles in L1 and L2 German
Source: Front Psychol. 2020 May 28;11:1045. doi: 10.3389/fpsyg.2020.01045 (PMC7270348; doi:10.3389/fpsyg.2020.01045)
Supplement: Supplementary file 1 [file Table_1.docx]

Supplementary Material

# Materials

List of items

| **participle forms** |  | **genuine adjectives** |  |
| --- | --- | --- | --- |
| bearbeitet | ‘processed’ | harmonisch | ‘harmonic’ |
| bestanden | ‘passed’ | gemütlich | ‘cozy’ |
| bestellt | ‘ordered’ | schlimm | ‘bad’ |
| empfohlen | ‘recommended’ | unbekannt | ‘unknown’ |
| erklärt | ‘explained’ | wichtig | ‘important’ |
| erlaubt | ‘permitted’ | langsam | ‘slow’ |
| fotografiert | ‘photographed’ | problematisch | ‘problematic’ |
| rasiert | ‘shaved’ | gruselig | ‘scary’ |
| repariert | ‘repaired’ | schädlich | ‘harmful’ |
| verbunden | ‘linked’ | glücklich | ‘happy’ |
| verliehen | ‘awarded’ | vorsichtig | ‘careful’ |
| verloren | ‘lost’ | praktisch | ‘useful’ |
| verschoben | ‘shifted’ | freundlich | ‘friendly’ |
| verstanden | ‘understood’ | langweilig | ‘tedious’ |
| zerrissen | ‘torn’ | schmutzig | ‘dirty’ |
| geändert | ‘changed’ | ehrlich | ‘honest’ |
| gebissen | ‘bitten’ | neidisch | ‘envious’ |
| gebügelt | ‘ironed’ | niedlich | ‘cute’ |
| gedruckt | ‘printed’ | fröhlich | ‘cheerful’ |
| gefunden | ‘found’ | zufrieden | ‘pleased’ |
| gefragt | ‘asked’ | gesund | ‘healthy’ |
| gegossen | ‘poured’ | glänzend | ‘shiny’ |
| gelernt | ‘learnt’ | positiv | ‘positive’ |
| gelesen | ‘read’ | trocken | ‘dry’ |
| geliebt | ‘loved’ | elegant | ‘elegant’ |
| geschlossen | ‘closed’ | kritisch | ‘critical’ |
| gespeichert | ‘saved’ | interessant | ‘interesting’ |
| gespielt | ‘played’ | aktuell | ‘up-to-date’ |
| gestochen | ‘stung’ | klassisch | ‘classical’ |
| getragen | ‘carried’ | spannend | ‘fascinating’ |
